# Bovine Collagen Peptides Compounds Promote the Proliferation and Differentiation of MC3T3-E1 Pre-Osteoblasts

**DOI:** 10.1371/journal.pone.0099920

**Published:** 2014-06-13

**Authors:** JunLi Liu, Bing Zhang, ShuJun Song, Ming Ma, ShaoYan Si, YiHu Wang, BingXin Xu, Kai Feng, JiGong Wu, YanChuan Guo

**Affiliations:** 1 Department of Pathology and Experimental Medicine, 306 Hospital of PLA, Beijing, People's Republic of China; 2 Key Laboratory of Photochemical Conversion and Optoelectronic Materials, Technical Institute of Physics and Chemistry, Chinese Academy of Science, Beijing, People's Republic of China; National Center for Scientific Research Demokritos, Greece

## Abstract

**Objective:**

Collagen peptides (CP) compounds, as bone health supplements, are known to play a role in the treatment of osteoporosis. However, the molecular mechanisms of this process remain unclear. This study aimed to investigate the effects of bovine CP compounds on the proliferation and differentiation of MC3T3-E1 cells.

**Methods:**

Mouse pre-osteoblast cell line MC3T3-E1 subclone 4 cells were treated with bovine CP compounds. Cell proliferation was analyzed by MTT assays and the cell cycle was evaluated by flow cytometry scanning. Furthermore, MC3T3-E1 cell differentiation was analyzed at the RNA level by real-time PCR and at the protein level by western blot analysis for runt-related transcription factor 2 (Runx2), a colorimetric p-nitrophenyl phosphate assay for alkaline phosphatase (ALP), and ELISA for osteocalcin (OC). Finally, alizarin red staining for mineralization was measured using Image Software Pro Plus 6.0.

**Results:**

Cell proliferation was very efficient after treatment with different concentrations of bovine CP compounds, and the best concentration was 3 mg/mL. Bovine CP compounds significantly increased the percentage of MC3T3-E1 cells in G_2_/S phase. Runx2 expression, ALP activity, and OC production were significantly increased after treatment with bovine CP compounds for 7 or 14 days. Quantitative analyses with alizarin red staining showed significantly increased mineralization of MC3T3-E1 cells after treatment with bovine CP compounds for 14 or 21 days.

**Conclusions:**

Bovine CP compounds increased osteoblast proliferation, and played positive roles in osteoblast differentiation and mineralized bone matrix formation. Taking all the experiments together, our study indicates a molecular mechanism for the potential treatment of osteoarthritis and osteoporosis.

## Introduction

Collagen peptides (CPs) are the hydrolysate components of collagen and are known to have efficacy against various pathologic conditions [1]–[2]. Regulatory agencies have generally recognized CP compounds as safe consumable products in pharmaceuticals and foods for a long time [3]. CP compounds from different species can produce a variety of active peptides with different bioactivities, such as high angiotensin I-converting enzyme inhibitory and antioxidant activities found in active peptides derived from fish or squid skin gelatins [4]–[5]. Two cryptic bioactive peptides, C2 (with cell adhesive properties) and E1 (with cell adhesive and antioxidant properties), have been isolated from bovine tendon collagen. The peptides supported faster wound closure than collagen under normal as well as stressed conditions [6]. Oral intake of specific bioactive CPs reduced skin wrinkles and had positive effects on dermal matrix synthesis [7]. Hydrolyzed collagen intake increased bone mass in growing rats trained with running exercise [8].

Osteoporosis increases bone fragility and susceptibility to fractures as a result of low bone mass and deterioration of the bone microarchitecture [9]. In recent years, CP compounds have been receiving scientific attention as potential oral supplements for the recovery of osteoarticular tissues. Osteoporosis rats treated with a collagen hydrolysate extracted from Sika deer velvet showed significant elevation of their bone mineral density levels compared with osteoporosis rats treated with retinoic acid [10]. A food supplement of hydrolyzed collagen improved the compositional and biodynamic characteristics of vertebrae in ovariectomized rats [11]. Administration of shark skin gelatin was able to increase the bone mineral density and type I collagen contents of ovariectomized rat femurs [12]. Oral administration of CP compounds improved the effects of calcitonin for postmenopausal osteoporosis patients [13]. Many patients with osteoarthritis or other types of arthritis have reported that their knee pain was reduced and other symptoms were significantly improved after treatment with 10 g/day of pharmaceutical-grade CP compounds administered orally for 6 months [14], indicating that CP compounds can potentially help osteoarthritis patients [15].

Although CP compounds, as bone health supplements, are known to play a role in the treatment of osteoporosis, the issue of whether bovine CP compounds promote the proliferation and differentiation of osteoblasts remains uncertain. MC3T3-EI pre-osteoblastic cells have the capacity to differentiate into osteoblasts and osteocytes and form calcified bone tissue *in vitro* [16]. In the present study, we first confirmed the effects of bovine CP compounds on the proliferation of MC3T3-E1 cells by MTT assays and cell cycle alterations by flow cytometry (FCM) scanning. We also analyzed the differentiation of MC3T3-E1 cells after treatment with CP compounds at the RNA level by real-time-PCR and at the protein level by western blot analysis for runt-related transcription factor 2 (Runx2), a colorimetric p-nitrophenyl phosphate assay for alkaline phosphatase (ALP), and ELISA for osteocalcin (OC). Finally, we examined the mineralization of MC3T3-E1 cells using alizarin red staining. We believe that our findings provide a molecular mechanism for the potential treatment of osteoarthritis and osteoporosis *in vitro*.

## Materials and Methods

### Preparation of MC3T3-E1 cells

Mouse pre-osteoblast cell line MC3T3-E1 subclone 4 cells (ATCC, Bethesda, MD, USA) [17] were obtained from the Chinese Medicine Academy of Sciences Cell Bank. The cells were maintained in maintenance medium consisting of high-glucose Dulbecco's modified Eagle's medium (hG-DMEM; Sigma-Aldrich, St. Louis, MO, USA) supplemented with 10% fetal bovine serum (FBS; Gibco, Carlsbad, CA, USA), 100 U/mL penicillin, and 100 U/mL streptomycin. When the cells became subconfluent, they were detached from the flask by treatment with an aqueous solution of 0.25% trypsin for 5 minutes, and the passaged cells were cultured in an incubator at 37°C under 5% CO_2_. For osteoblast differentiation, the cells were induced with osteogenic medium (10 mmol/L β-glycerophosphate, 10 nmol/L dexamethasone, and 50 mg/L ascorbic acid-2-phosphate) and the osteogenic medium was changed every 3 days.

### Determination of the molecular weight distributions of bovine CP compounds by MALDI-TOF-MS

CP compounds from bovine bone (Dongbao Biotechnology Co. Ltd., Baotou, China) were used for our experiments. MALDI-TOF-MS analyses showed that the bovine CP compounds produced high intensity matrix peaks at the *m/z* value range of 0.6–2.5 kDa, with relatively higher abundances mainly at 0.66, 0.88, 1.1, and 1.44 kDa, and other combinations, most of them with *m/z* values above 1.5 kDa.

### Cell proliferation assessment by the MTT assay

Cell proliferation was measured using a standard methyl thiazolyl tetrazolium (MTT) assay (Sigma-Aldrich). MC3T3-E1 cells were treated with bovine CP compounds at concentrations of 0–6 mg/mL in hG-DMEM with 10% FBS. MC3T3-E1 cells after the third passage were seeded in 96-well plates at a density of 3×10^3^ cells/well with six duplicate wells per treatment, and cultured for 3, 5, and 7 days with the above CP concentrations. MTT reagent (5 mg/mL) was added to each well and incubated for 4–6 hours. Each mixture was then carefully removed and 150 µL of dimethyl sulfoxide (Sigma-Aldrich) was added to each well by pipetting up and down several times. The plates were kept on a rocker shaker for 10 minutes at room temperature, and the absorbances were measured at 490 nm with a microplate reader.

### Cell cycle analysis by FCM scanning

Cell cycle analyses were evaluated by the DNA distribution and examined using propidium iodide (PI) staining (Sigma-Aldrich). Briefly, MC3T3-E1 cells were harvested after 4 or 5 days of treatment with 3 mg/mL bovine CP compounds or no treatment (CN cells), fixed in 70% chilled ethanol, and kept at −20°C for at least 24 hours. To measure the DNA contents, the cells were washed twice with PBS, digested with 10 µg/mL RNaseA (Sigma-Aldrich) for 10 minutes, and then stained with 50 µg/mL PI. The DNA contents were analyzed using a flow cytometer (Becton Dickinson, Mountain View, CA, USA).

### Evaluation of typical bone-related gene expressions by real-time PCR

MC3T3-E1 cells were seeded in 6-well plates at a density of 8×10^4^ cells/well, and treated with osteogenic medium. The RNA levels were analyzed by real-time PCR in cells treated with 3 mg/mL bovine CP compounds and untreated (CN) cells for 7 or 14 days. Briefly, total RNA was extracted using TRIzol reagent (Invitrogen) and the purified total RNA was used for cDNA synthesis with M-MLV reverse transcriptase (Promega, Madison, WI, USA) and oligo(dT) primers. The specific primers used for detecting the mRNA transcripts of the Runx2, Col1α1, ALP, OC, and β-actin genes are shown in Table 1. The transcript levels were normalized by the β-actin transcript levels. The PCR conditions were: 95°C for 3 minutes; and 40 cycles of 94°C for 10 seconds and 60°C for 30 seconds.

**Table 1:** Targeted gene sequences of the primers used for real-time PCR.

Gene Accession No. Primers (F = forward; R = reverse)		
Runx2	NM_001145920.	F: 5′-CCCTGAACTCTGCACCAAGT-3′
		R: 5′-TGGAGTGGATGGATGGGGAT-3′
ALP	NM_016798	F: 5′-TGAATGACGGGCCTGATGAC-3′
		R: 5′-GGTACTTATCCCGGGCCTTG-3′
OC	NM_007541	F: 5′-TGACCTCACAGATGCCAAGC-3′
		R: 5′-CGCCGGAGTCTGTTCACTAC-3′
Col1α1	NM_007742	F: 5′-CCAGCCGCAAAGAGTCTACA-3′
		R: 5′-TTCCACGTCTCACCATTGGG-3′
β-actin	NM_001281595	F: 5′-TTCGTTGCCGGTCCACACCC-3′
		R: 5′-GCTTTGCACATGCCGGAGCC-3′

Runx2: runt-related transcription factor 2; ALP: alkaline phosphatase; Col1α1: type I collagen; OC: osteocalcin.

### Western blot analysis

MC3T3-E1 cells were collected after 7 or 14 days of treatment with 3 mg/mL bovine CP compounds or no treatment (CN cells). The cells were lysed in lysis buffer containing protease inhibitors and 15-μg aliquots were subjected to western blot analysis using a specific primary antibody against Runx2 and a secondary antibody linked with horseradish peroxidase (Boston Biotechnology, Wuhan, China). An anti-β-actin antibody (Santa Cruz Biotechnology, Santa Cruz, CA, USA) was used for β-actin evaluation as an internal control. The bound antibodies were detected with an enhanced chemiluminescent detection reagent (Pierce Biotechnology Inc., Rockford, IL, USA), and the intensities of the bands were measured using Image Software Pro Plus 6.0 software.

### ALP analysis using a colorimetric p-nitrophenyl phosphate assay

ALP is an enzyme expressed by cells, and its activity is a well-defined marker for osteogenesis. A colorimetric p-nitrophenyl phosphate assay was used to measure the expression of ALP in osteoblasts. After 7 or 14 days of culture, MC3T3-E1 cells were washed with PBS and lysed with 0.1% Triton X-100 using three cycles of freezing and thawing to verify that the cells were completely lysed. The cell lysates were centrifuged at 15,000×*g* for 5 minutes at 4°C, and the supernatants were collected. Next, 5-μL aliquots of the cell lysates were transferred to 96-well plates and 150 µL of p-nitrophenyl phosphate (Jiancheng Biotechnology, Nanjing, China) was added to each well as a substrate for 20 minutes at 37°C. p-Nitrophenyl phosphate was quantified based on the spectrophotometric absorbance at 405 nm. The ALP activity was normalized by the total protein concentration for each sample using a BCA protein assay (Pierce Biotechnology Inc.).

### OC analysis by ELISA

The OC levels were measured in cell culture medium using a Mouse Osteocalcin ELISA Kit (Yueyan Biotechnology, Shanghai, China) according to the manufacturer's protocol. Briefly, 50 µL of standard, 40 µL of sample, 10 µL of anti-OC antibody, and 50 µL of streptavidin-horseradish peroxidase reagent were added to 96-well ELISA plates and incubated at 37°C for 1 hour. The wells were then washed five times with washing buffer. Next, 50 µL of Chromogen Solution A and 50 µL of Chromogen Solution B were added to each well and incubated at 37°C for 10 minutes in the dark. Finally, 50 µL of TMB solution was added and the absorbances were measured at 450 nm with a colorimetric microplate reader.

### Mineralization analysis by alizarin red staining

To measure extracellular matrix mineralization deposits for bone nodule formation, the cellular matrix was stained using alizarin red dye that combines with Ca^2+^ in the matrix [18]. Red staining with alizarin red is an indicator of mineralization. Briefly, MC3T3-E1 cells were seeded on 24-well plates, and induced with osteogenic medium after being treated with 0.3 or 3 mg/mL bovine CP compounds or left untreated (CN cells) for 14 or 21 days. The cells were washed twice with PBS, fixed with 4% paraformaldehyde for 30 minutes at 4°C, and rinsed with deionized water. Finally, the cells were stained with 40 mmol/L alizarin red solution (pH 4.4) for 5–10 minutes at room temperature and rinsed twice with deionized water. Images of the stained cells were captured using a digital camera (IM50; Leica, Jena, Germany). Quantitative analyses of the mineralization indicated by alizarin red staining were performed using Image Software Pro Plus 6.0.

### Statistical analysis

Statistical analyses were performed using SPSS-17.0 software (SPSS Inc., Chicago, IL, USA). All data were analyzed by analysis of variance (ANOVA) and Student's *t-*test, and differences were considered statistically significant for values of *p*<0.05. All experiments were carried out independently at least three times unless indicated otherwise.

## Results

### Effects of bovine CP compounds on the proliferation of MC3T3-E1 cells

**Figure 1:**
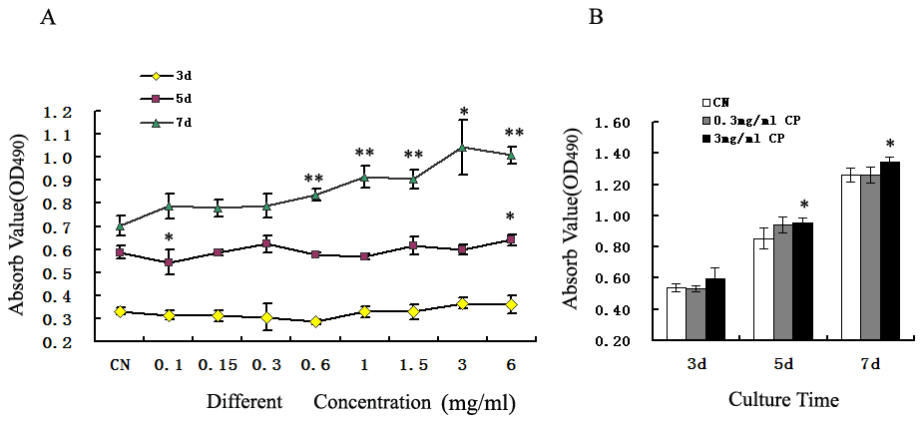
Effects of bovine CP compounds on the proliferation of MC3T3-E1 cells. The proliferation of MC3T3-E1 cells was measured by MTT assays after treatment with CP at 0.1–6 mg/mL (A) or 0.3 and 3 mg/mL (B) for 3–7 days. Data are expressed as means ± standard deviation (*n* = 6). *p<0.05, **p<0.01, Significant difference from CN. CP: bovine collagen peptides compounds; CN: control.

The colorimetric MTT assay is a main application that allows the proliferation of cells to be assessed. MC3T3-E1 cells were cultured after treatment with 0.1–6 mg/mL bovine CP compounds for 3, 5, and 7 days. No significant differences were observed between the optical density (OD) values of the CN and CP compound-treated cells at 3 days. After 5 days, the OD values of the CP compound-treated cells were slightly lower at 0.1 mg/mL, but higher at 6 mg/mL, compared with those of the CN cells (*p*<0.05). The OD values of the MC3T3-E1 cells after treatment with 0.6–6 mg/mL bovine CP compounds for 7 days were much higher than those of the CN cells (*p*<0.01, Figure 1A), and the best concentration of bovine CP compounds for proliferation at 7 days was 3 mg/mL (*p*<0.01, Figure 1B). Therefore, the concentration of 3 mg/mL bovine CP compounds was selected for further experiments.

### Alteration of the cell cycle distribution of MC3T3-E1 cells by bovine CP compound treatment

**Figure 2:**
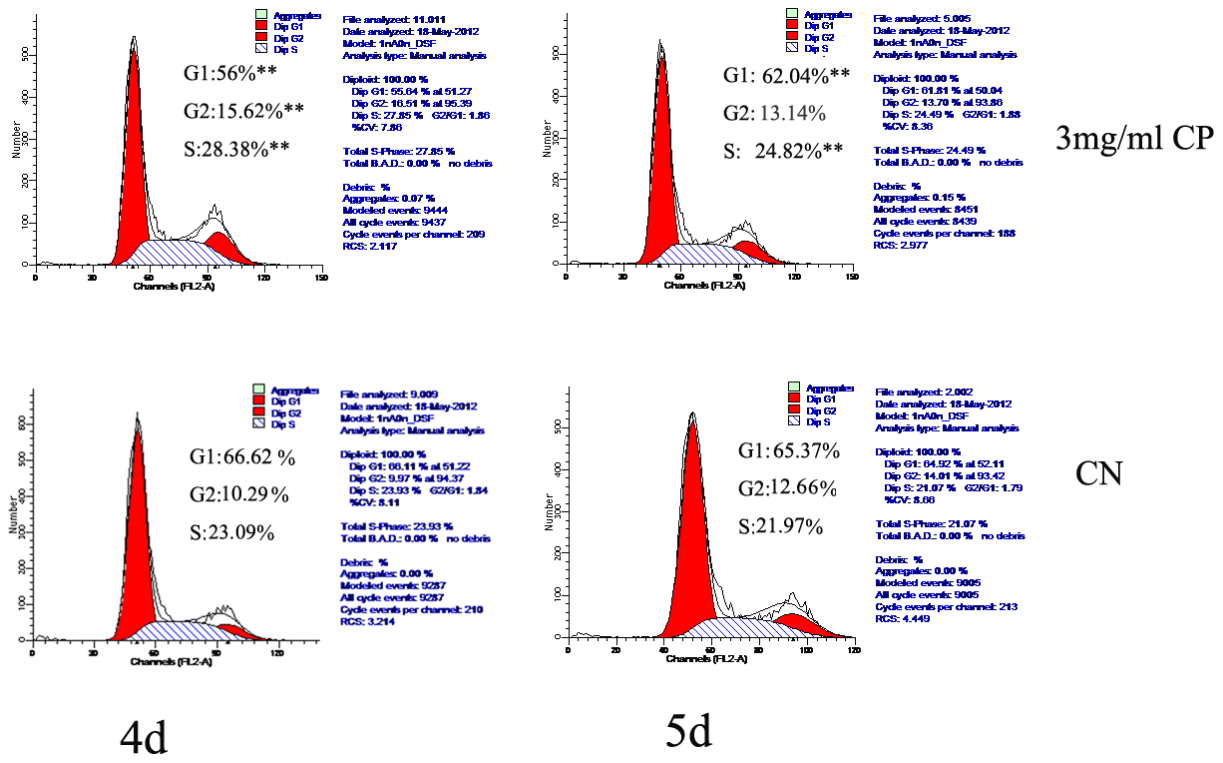
Alteration of the cell cycle distribution of MC3T3-E1 cells by bovine CP compound treatment. Cell cycle analyses were performed by FCM scanning. Representative histograms of MC3T3-E1 cells in the CP-treated and CN groups at 4–5 days are shown. Data are expressed as means ± standard deviation (n = 4). *p<0.05, **p<0.01, Significant difference from CN. CP: bovine collagen peptides compounds; CN: control.

We further investigated whether bovine CP compounds affected the cell cycle of MC3T3-E1 cells. Following PI staining, cell cycle distribution analyses of MC3T3-E1 cells were conducted by FCM scanning. As shown in Figure 2, treatment with 3 mg/mL bovine CP compounds led to increased cell cycle arrest in G_2_/S phase at 4 or 5 days (*p*<0.01), and G_1_ phase was significantly reduced compared with the CN cells (*p*<0.01).

### Real-time-PCR analysis of the expressions of different osteoblast markers after bovine CP compound treatment

**Figure 3:**
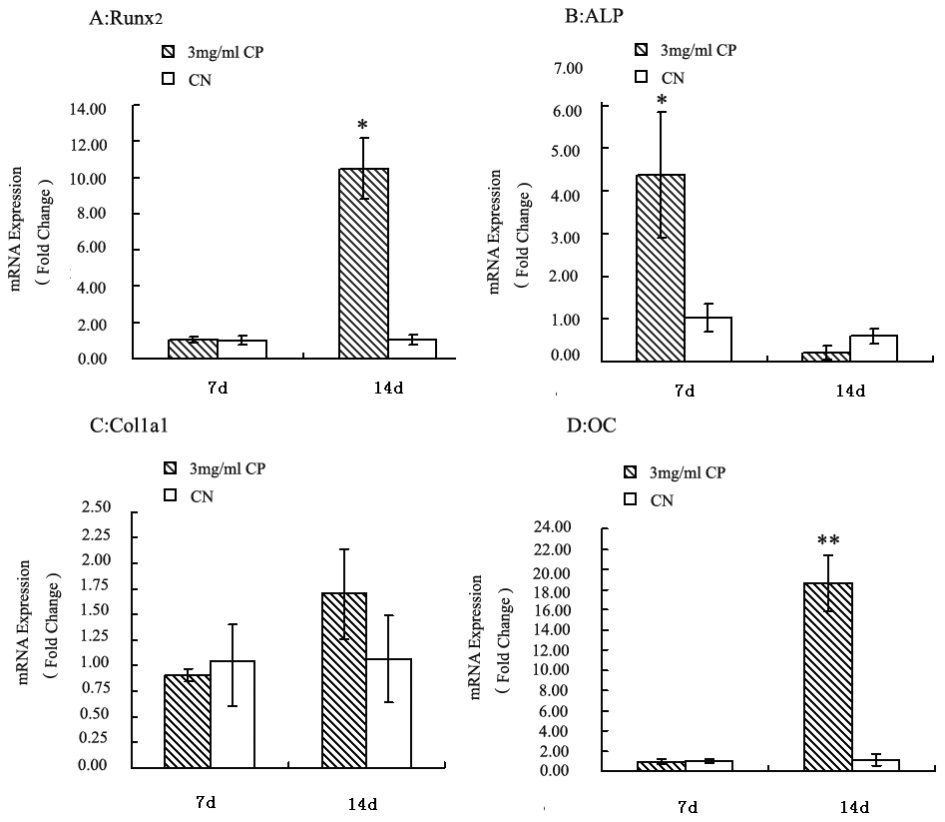
Real-time-PCR analysis of the expressions levels of different osteoblast markers after bovine CP compound treatment. Real-time PCR analyses of the Runx2 (A), ALP (B), Col1α1 (C), and OC (D) mRNA levels in MC3T3-E1 cells induced with osteogenic medium after treatment with 3 mg/mL CP or no treatment (CN) for 7 or 14 days are shown. The results were normalized by the mRNA levels of β-actin as a housekeeping gene. Data are expressed as means ± standard deviation (*n* = 4). *p<0.05, **p<0.01, Significant difference from CN. CP: bovine collagen peptides compounds; CN: control.

To gain further insights into the molecular mechanisms of the bovine CP compound functions in osteoblast differentiation, the expressions of typical bone-related genes were examined by real-time PCR. The expression level of Runx2, which regulates osteoblast differentiation at early stages, was markedly upregulated in the bovine CP compound-treated cells at 14 days compared with the CN cells (*p*<0.05, Figure 3A). The ALP mRNA expression level in the CP compound-treated cells was significantly higher at 7 days (*p*<0.05, Figure 3B) and then decreased at 14 days compared with the CN cells. The Col1α1 mRNA levels in the bovine CP compound-treated cells were not significantly increased compared with those in the CN cells at 7 or 14 days (*p*>0.05, Figure 3C). OC mRNA expression was rapidly increased at 14 days (*p*<0.05, Figure 3D) in the CP compound-treated cells.

### Effects of bovine CP compounds on the ALP activity in MC3T3-E1 cells

**Figure 4:**
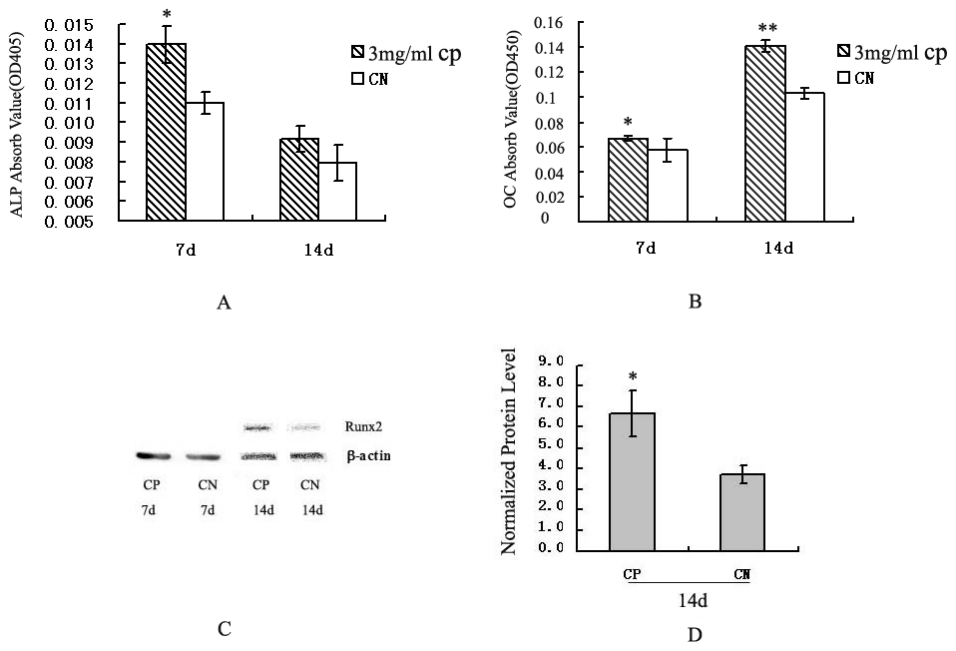
Osteogenic activities of bovine CP compounds in MC3T3-E1 cells. The ALP activity (A), OC amount (B), and Runx2 expression (C, D) were determined by colorimetric analysis, ELISA, and western blotting analysis, respectively. MC3T3-E1 cells induced with osteogenic medium after treatment with 3 mg/mL CP or no treatment (CN) for 7 or 14 days are shown. The expression of Runx2 was normalized by that of β-actin as a housekeeping gene (C, D). Data are expressed as means ± standard deviation (*n* = 3). *p<0.05, **p<0.01, Significant difference from CN. CP: bovine collagen peptides compounds; CN: control.

Next, we evaluated whether the bovine CP compounds enhanced ALP activity in MC3T3-E1 cells. MC3T3-E1 cells were cultured with osteogenic medium for 7 or 14 days. The ALP activity in the bovine CP compound-treated cells was much higher than that in the CN cells at 7 days (*p*<0.05, Figure 4A), but no significant difference was observed between the CN and CP compound-treated cells at 14 days. These results were consistent with the real-time PCR analyses described above, and indicate that bovine CP compounds can promote ALP activity in MC3T3-E1 cells at the early stage.

### Effects of bovine CP compounds on the OC amount in MC3T3-E1 cells

In parallel, the OC levels in the supernatants were measured by ELISA. The OC level in the bovine CP compound-treated cells was significantly higher than that in the CN cells at 14 days (*p*<0.05, Figure 4B), but there was no significant difference at 7 days. These results indicate that bovine CP compounds can promote the OC level in MC3T3-E1 cells at a late stage.

### Effects of bovine CP compounds on Runx2 expression in MC3T3-E1 cells

The expression of Runx2 was examined by western blot analysis. No expression of Runx2 was detected in the CN and bovine CP compound-treated cells at 7 days. However, the expression of Runx2 was markedly upregulated in the bovine CP compound-treated cells at 14 days compared with the CN cells (*p*<0.05, Figure 4C, 4D).

### Effects of bovine CP compounds on mineralization in MC3T3-E1 cells

**Figure 5:**
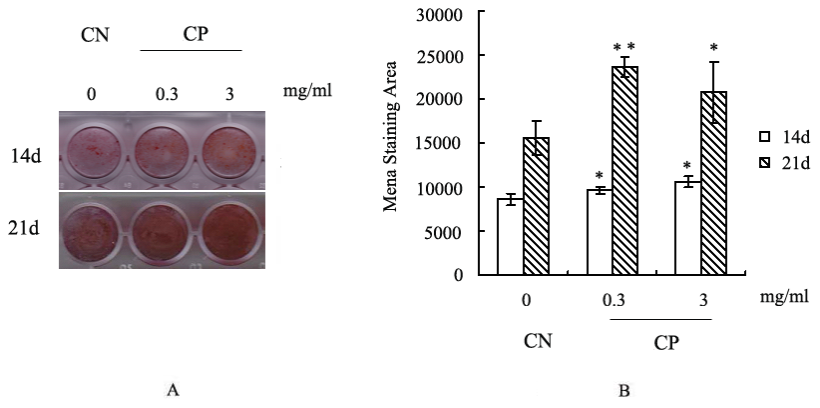
Influence of bovine CP compounds on mineralization in MC3T3-E1 cells. The calcium deposits in the mineralized matrix were analyzed by alizarin red staining. MC3T3-E1 cells induced with osteogenic medium after treatment with 0.3–3 mg/mL CP or no treatment (CN) for 14 or 21 days are shown. Data are expressed as means ± standard deviation (*n* = 4). *p<0.05, Significant difference from CN. CP: bovine collagen peptides compounds; CN: control.

Mineralization is important for bone formation. To further examine the importance of bovine CP compounds for bone mineralization, quantitative assays of mineralization were carried out using alizarin red staining. The presence of calcium deposits showed that treatment with 0.3 and 3 mg/mL bovine CP compounds significantly increased the mineralization in MC3T3-E1 cells at 14 and 21 days compared with the CN cells (*p*<0.05, Figure 5A, 5B). These data indicate that bovine CP compounds can promote mineralization in MC3T3-E1 cells.

## Discussion

This study has demonstrated for the first time that CP compounds with molecular weights of 0.6–2.5 kDa isolated from bovine bone can promote osteoblast proliferation and differentiation *in vitro*. In the present study, we first found that bovine CP compounds markedly increased osteoblast growth in a dose-dependent manner. This result is consistent with a recent report that collagen hydrolysate (CH) with molecular weights of <3 kDa isolated from porcine skin gelatin stimulated the proliferation of human osteoblastic cell line MG63 cells [19]. These findings indicate that the bone-protective effects of CP compounds from different species partly arise through osteoblast proliferation. Therefore, the question remains as to the exact role and mechanism by which bovine CP compounds impart this regulatory control on osteoblast proliferation, because the correct cell cycle regulated by a series of cell cycle control proteins is an important guarantee of cell proliferation [20], and bovine CP compound treatment led to increased cell cycle arrest in G_2_/S phase with prolonged incubation time. We concluded that bovine CP compounds can affect the proliferation of MC3T3-E1 cells by promoting DNA synthesis.

Furthermore, MC3T3-E1 cells were used to investigate the influence of CP compounds on the regulation of osteoblast-associated genes, namely Runx2, ALP, Col1α1, and OC. These genes are major phenotypic markers for pre-osteoblast differentiation during bone formation [21]. During early stages of osteoblast differentiation, osteoblasts synthesize Col1α1 and other matrix proteins, followed by the production of ALP and other osteoblastic differentiation markers, ultimately leading to the induction of extracellular matrix calcification [22]–[23].

Runx2 determines osteoblast differentiation at early stages from multipotent mesenchymal cells [24] and is a master regulatory gene in bone formation. Mutations in Runx2 have been identified in patients with cleidocranial dysplasia, which is a rare autosomal dominant skeletal dysplasia characterized by delayed closure of the cranial sutures [25], and Runx2 has been shown to regulate the expressions of type I collagen and OC [26]. In this study, we found that both the mRNA and protein levels of Runx2 were increased in the bovine CP compound-treated cells compared with the CN cells, demonstrating that CP compounds promoted the differentiation of osteoblasts by upregulating the level of Runx2 expression.

ALP is a marker of bone formation and differentiation of osteoblasts [27]. It hydrolyzes pyrophosphate and provides inorganic phosphate to promote mineralization in osteoblasts [28]. Our results showed that ALP activity was detected in the early stages and increased concomitantly with osteoblast differentiation. OC is the most abundant protein in the bone matrix, and is essential for hydroxyapatite binding and deposition in the extracellular matrix of bone [29]. Our findings suggested that bovine CP compounds significantly increased the amount of OC at a late stage. Most importantly, bovine CP compounds significantly promoted the formation of mineralized nodules in MC3T3-E1 cells. Taken together, these results suggest that bovine CP compounds exerted a dominant effect on the stimulation of bone formation.

Col1α1 is the most abundant protein synthesized by active osteoblasts and is essential for mineral deposition, and its expression therefore represents the start of osteoblast differentiation [30]. As bone health supplements, bovine CP compounds are thought to stimulate increased synthesis of collagen [31]. Mitogen-activated protein kinases and extracellular signal-regulated kinases (ERKs) play important roles in osteoblast proliferation and differentiation [32]–[33]. Recently, it was reported that treatment with CH from porcine skin gelatin significantly increased the collagen content and expression of the Col1α1 gene through phosphorylation of ERK1/2 [19]. However, at the molecular level, we found that the expression of Col1α1 mRNA was not significantly upregulated after treatment with bovine CP compounds, which is consistent with a report that none of the collagen hydrolysates obtained from different sources stimulated the biosynthesis of collagen [6]. To address this, future studies are warranted to determine whether different concentrations of bovine CP compounds can influence Col1α1 expression, and the resulting data will certainly provide more insights into the nature of the collagen produced.

In conclusion, our data have demonstrated for the first time that bovine CP compounds not only increased osteoblast proliferation, but also appeared to have positive roles in osteoblast differentiation and mineralized bone matrix formation. From this point of view, we have probably provided a reasonable basis for the potential utility of bovine CP compounds in the prevention and treatment of osteoporosis. In a follow-up study, we will continue to study the mechanism underlying osteoporosis prevention and treatment by bovine CP compounds in ovariectomized rats.
